# Circulating microparticles are prognostic biomarkers in advanced non-small cell lung cancer patients

**DOI:** 10.18632/oncotarget.18372

**Published:** 2017-06-06

**Authors:** Chin-Chou Wang, Chia-Cheng Tseng, Huang-Chih Chang, Kuo-Tung Huang, Wen-Feng Fang, Yu-Mu Chen, Cheng-Ta Yang, Chang-Chun Hsiao, Meng-Chih Lin, Chi-Kung Ho, Hon-Kan Yip

**Affiliations:** ^1^ Division of Pulmonary and Critical Care Medicine, Department of Internal Medicine, Kaohsiung Chang Gung Memorial Hospital, Chang Gung University College of Medicine, Kaohsiung, Taiwan; ^2^ Department of Public Health, Kaohsiung Medical University, Kaohsiung, Taiwan; ^3^ Department of Respiratory Care, Chang Gung University of Science and Technology, Chiayi Campus, Chiayi, Taiwan; ^4^ Graduate Institute of Clinical Medical Sciences, Chang Gung University College of Medicine, Kaohsiung, Taiwan; ^5^ Department of Pulmonary and Critical Care Medicine, Chang Gung Memorial Hospital, Chang Gung University College of Medicine, Taoyuan, Taiwan; ^6^ Center for Shockwave Medicine and Tissue Engineering, Kaohsiung Chang Gung Memorial Hospital and Chang Gung University College of Medicine, Kaohsiung, Taiwan; ^7^ Division of cardiology, Department of Internal Medicine, Kaohsiung Chang Gung Memorial Hospital and Chang Gung University College of Medicine, Kaohsiung, Taiwan; ^8^ Department of Medical Research, China Medical University Hospital, China Medical University, Taichung, Taiwan; ^9^ Department of Nursing, Asia University, Taichung, Taiwan; ^10^ Institute for Translational Research in Biomedicine, Kaohsiung Chang Gung Memorial Hospital, Kaohsiung, Taiwan

**Keywords:** advanced non-small cell lung cancer, microparticles, disease control, disease progression

## Abstract

We investigated whether circulating microparticles (MPs) could serve as prognostic biomarkers in non-small cell lung cancer (NSCLC) patients. We enrolled 25 control subjects and 136 NSCLC patients categorized into disease-progression (DP, n=42) and disease-control (DC, n=94) groups. Flow cytometric analysis showed that levels of four types of circulating microparticles (EDAc-MPs, EDAp-MPs, PDAc-MPs and PDAp-MPs) were higher in the study patients than the control subjects (*P* < 0.04). DP patients showed poor initially performance status and more non-adenocarcinomas than DC patients. DC patients showed more EGFR mutations and poorer performance to targeted therapy than DP patients (*P* < 0.01). Three months after therapy, the levels of all four types of circulating MPs were lower in DC than DP patients (*P* < 0.02), and were comparable to the levels in control subjects. In addition, the levels of circulating MPs after 3 months accurately predicted one-year prognostic outcomes (*P* < 0.05). This study showed that circulating MPs are valuable prognostic biomarkers in advanced NSCLC patients.

## INTRODUCTION

Advanced lung cancer (LC) is a leading cause of cancer deaths worldwide [1–4]. Nearly 95% of all lung cancers are either small cell lung cancer (SCLC) or non-small cell lung cancer (NSCLC). Current treatments include a combination of traditional surgical interventions and adjunctive radiation and chemotherapy. Molecularly targeted drugs for LC include epidermal growth factor receptor tyrosine kinase inhibitors (EGFR TKI) like gefitinib, erlotinib, and afatinib [5–9], and anaplastic lymphoma kinase tyrosine kinase inhibitors (ALK TKI) like crizotinib [10]. However, the overall long-term survival rate from lung cancer is extremely low [11–14]. Despite advanced technology, nearly 50% of lung cancer patients are diagnosed at an advanced stage [4]. Thus, better understanding of the lung cancer pathogenesis and development of effective molecular and cellular biomarkers [4] are necessary to detect cancer early and improve therapeutic outcomes [15–17]. The development of serum biomarkers like microparticles would be useful to predict prognostic outcomes in LC [18–21].

Microparticles (MPs) or membrane-bound vesicles are small fragments of the plasma membrane released by activated and/or apoptotic cells. The MPs ranging from 0.1 to 1.0μm in size circulate in blood and other body fluids and are known to mediate inflammation and thrombosis [22–31]. Additionally, MPs have differential effects on angiogenesis depending on their origin [22, 26, 28–31]. Microparticles from platelets promote capillary network formation and production of pro-angiogenic factors [22, 24, 28, 30, 32]. In contrast, both endothelial- and lymphocyte-derived MPs possess either pro- or anti-angiogenic properties depending on the stimuli [28, 29].

Circulating MPs are also associated with a wide range of diseases including LC [28, 32–39]. Circulating endothelial-derived activated MPs (EDAc-MPs) were useful in predicting 1-year morality in advanced stage NSCLC patients [20]. However, since majority of the patients had received palliative treatment prior to enrolment in our previous study, the PDAp-MPs (platelet-derived apoptotic MPs), PDAc-MPs (platelet-derived activated MPs), and EDAp-MPs (endothelial-derived apoptotic MPs) were not prognostic [20]. Therefore, we conducted this prospective study by measuring the circulating levels of MPs in advanced stage NSCLC patients to analyze their prognostic outcomes in advanced stage NSCLC patients.

## RESULTS

### Baseline circulating levels of four types microparticles in study subjects

**Figure 1:**
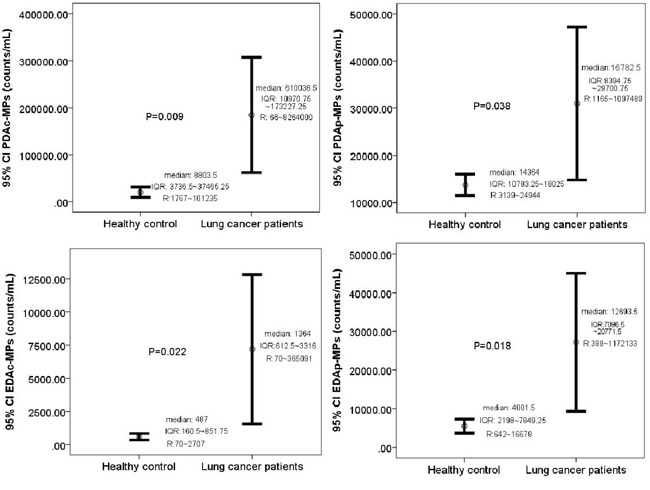
Comparison of baseline levels of circulating microparticles in the study and control subjects Circulating levels of **(A)** Platelet-derived activated MPs (PDAc-MPs; p = 0.009), **(B)** Platelet-derived apoptotic MPs (PDAp-MPs; p = 0.038), **(C)** Endothelial-derived activated MPs (EDAc-MPs; p = 0.022) and **(D)** Endothelial-derived apoptotic MPs (EDAp-MPs; p = 0.018) in study and control subjects. Note: CI = confidence intervals.

The circulating levels of the PDAc-MPs, PDAp-MPs, EDAc-MPs and EDAp-MPs were significantly higher in advanced NSCLC patients compared to control subjects. This suggested that the circulating MPs are useful diagnostic biomarkers for advanced NSCLC patients (Table 1 and Figure 1).

**Table 1:** Comparison of circulating levels of four types microparticles between lung cancer patients and healthy control group

Variables	Study Group (n=136)*	Control Group (n=25)	P-value
PDAc-MPs	184866.40 ± 723526.40	20334.75 ± 25884.62	0.009
PDAp-MPs	30988.10 ± 95402.90	13725.08 ± 5401.12	0.038
EDAc-MPs	7196.16 ± 33141.41	598.54 ± 582.43	0.022
EDAp-MPs	27171.17 ± 105285.34	5486.17 ± 4331.44	0.018

Data are expressed as mean ± SD.

PDAc-MPs = platelet-derived activated microparticles; PDAp-MPs = platelet-derived apoptotic MPs; EDAc-MPs = endothelial-derived activated MPs; EDAp-MPs = endothelial-derived apoptotic MPs.

* indicated the blood sample was drawn prior to treatment.

### Baseline characteristics of study patients

The disease control (DC) and disease progression (DP) groups had similar parameters like gender, weight, height, surface area, mass index and incidence of smoking status. The serum levels of total cholesterol, sugar, glutamic oxaloacetic transaminase (GOT) and glutamic pyruvic transaminase (GPT) were also similar between these two groups. Furthermore, the red blood cell (RBC), white blood cell (WBC) and platelet counts as well as circulating levels of carcinoembryonic antigen (CEA) were also similar between the 2 groups. Furthermore, comorbidities like hypertension, diabetes mellitus, coronary artery disease and chronic obstructive lung disease were comparable between DC and DP groups (Table 2).

**Table 2:** Baseline Characteristics of 136 Study Patients

Variable	Disease Progression (n=42)	Disease Control (n=94)	P-value
Age	62.69 ± 10.80	65.01 ± 10.23	0.244
Sex (male)	64.3% (27)	61.7% (58)	0.849
Body weight (kg)	65.47 ± 19.72	73.04 ± 35.09	0.112
Body height (cm)	162.45 ± 23.02	147.34 ± 36.17	0.004
Body surface area (m^2^)	1.67 ± 0.18	1.64 ± 0.18	0.338
Body mass index (kg/m^2^)	23.61 ± 3.27	23.31 ± 3.66	0.642
Smoking status	50% (21)	54.2% (51)	0.711
Total cholesterol (mg/dL)	176.33 ± 49.09	182.25 ± 45.51	0.506
Triglyceride (mg/dL)	178.75±81.92	193.12±86.47	0.365
Ac sugar (mg/dL)	123.17 ± 48.15	128.36 ± 66.16	0.608
Creatinine	0.90±0.34	0.89±0.35	0.928
Na	136.69±8.20	137.10±6.91	0.766
K	3.99±0.59	3.85±0.57	0.224
Aspartate aminotransferase (IU)	26.38 ± 16.36	25.62 ± 12.22	0.766
Alanine aminotransferase (IU)	31.14 ± 38.10	28.79 ± 27.36	0.685
White blood cell count (x10^3^/mL)	8.23±5.20	7.92±3.48	0.690
Red blood cell count (x10^6^/mL)	4.41±0.58	4.44±0.73	0.814
Platelet count (x10^3^/mL)	27.7±12.7	26.4±10.5	0.539
CEA	357.16±1760.44	75.88±166.13	0.302
Underlying comorbidity			
Hypertension	47.6% (20)	39.4% (37)	0.452
Diabete mellitus	19% (8)	18.1% (17)	1.000
COPD	9.5% (4)	12.1% (11)	1.000
CAD	14.3% (6)	22.3% (21)	0.355

Data are expressed as mean ± SD or % (n).

### Lung cancer associated parameters in the study patients

The cell types of lung cancer (adenocarcinoma or non-adenocarcinoma) were similar between the DC and the DP groups. However, higher epidermal growth factor receptor (EGFR) mutation levels were observed in the DC patients compared to DP group. The incidence of metastasis and the metastatic sites were similar for the two groups of the patients. Also, the two groups showed no differences in stages IIIB or IV. However, the DP patients’ performance status was poorer than the DC group, and the DC group of patients was easily met with target therapy (Table 3).

**Table 3:** Lung Cancer Associated Parameters in 136 Study Patients

Variables	Disease Progression (n=42)	Disease Control (n=94)	P-value
Cell type			0.049
Adenocarcinoma	73.8% (31)	87.2% (82)	
Non-adenocarcinoma	26.2% (11)	12.8% (12)	
Metastasis			0.355
M0	23.8% (10)	21.3% (20)	
M1a	23.8% (10)	36.2% (34)	
M1b	52.4% (22)	42.5% (40)	
Stage			0.824
IIIb	23.8% (10)	21.3% (20)	
IV	76.2% (32)	78.7% (74)	
Metastatic site			
Pleura	31% (13)	41.5% (39)	0.259
Lung	40.5% (17)	26.6% (25)	0.440
Bone	28.6% (12)	34% (32)	0.559
Liver	7.1% (3)	8.5% (8)	1.000
Adrenal gland	7.1% (3)	1.1% (1)	0.087
Brain	16.7% (7)	12.8% (12)	0.596
Performance status			0.008
0	14.3% (6)	9.6% (9)	
1	57.1% (24)	80.9% (76)	
2	28.6% (12)	9.6% (9)	
Therapeutic Intervention			0.000
Target therapy	11.9% (5)	70.2% (66)	
Chemotherapy	88.1% (37)	29.8% (28)	
EGFR status	11.9% (5)	70.2% (66)	0.000

Data are expressed as % (n).

### Circulating microparticle levels in DC and DP patients

**Figure 2:**
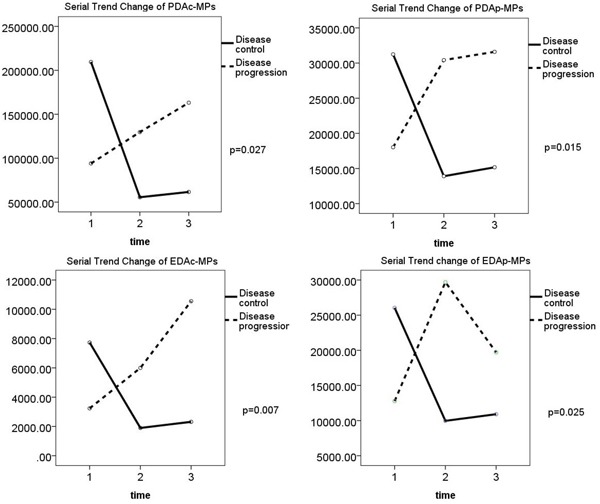
Comparison of changes in levels of the four types of microparticles in disease control (DC) and disease progression (DP) groups Changes in levels of **(A)** PDAc-MPs (p=0.027), **(B)** PDAp-MPs (p=0.015), **(C)** EDAc-MPs (p=0.007) and **(D)** EDAp-MPs (p=0.025) between DC and DP group of patients.

Table 4 shows the changes in circulating levels of PDAc-MPs, PDAp-MPs, EDAc-MPs, EDAp-MPs between the DC and DP patients prior to and at the end of 1^st^ and 3^rd^ months after pharmacological intervention. The circulating levels of the four types of MPs were similar between the DC and DP groups prior to and at the end of the first month after pharmacological therapy (Figure 2). However, at the end of the third month after pharmacological therapy, all the four types of MPs were significantly lower in the DC group than in the DP group (Figure 2). This was corroborated by positive ΔEDAp-MP, ΔEDAc-MP, ΔPDAp-MP and ΔPDAc-MP values associated with DP. These findings demonstrated that the pharmacological therapy lowered the circulating MPs in the DC group (Table 4 and Figure 2).

**Table 4:** Serial changes of circulating microparticles between disease progression and disease control patients

Variables	Disease Progression (n=42)	Disease Control (n=94)	P-value
PDAc-MPs (time 1)*			0.554
mean±SD	129713.69 ± 181619.98	209509.10 ± 862192.46	
median	19720.5	11749.5	
interquartile range	6978.75~29012.25	7050.25~18300	
range	3307~408654	399~1172133	
PDAp-MPs (time 1)*			0.963
mean±SD	30424.81 ± 36668.18	31239.79 ± 112335.20	
median	1290.0	1432	
interquartile range	672.25~3950	603.5~3220	
range	160~92792	70~365091	
EDAc-MPs (time 1)*			0.779
mean±SD	5998.31 ± 15338.89	7731.37 ± 38596.88	
median	20198.5	15393	
interquartile range	8434.25~38064	8244.25~25611	
range	1042~8637.5	1165~1097489	
EDAp-MPs (time 1)*			0.816
mean±SD	29695.21 ± 62524.79	26043.40 ± 119847.80	
median	54283.5	26465	
interquartile range	9166.75~193062.75	12291.75~69015.5	
range	1042~863715	66~8264000	
PDAc-MPs (time 2)†			0.267
mean±SD	61578.98 ± 83592.25	94027.07 ± 106312.33	
median	10621	11047	
interquartile range	7148.5~14540	6145.75~18297.5	
range	1818~26138	134~46044	
PDAp-MPs (time 2)†			0.325
mean±SD	15173.62 ± 11688.99	18028.77 ± 17020.22	
median	763.5	2240.5	
interquartile range	423.25~1791.75	765~4536.75	
range	104~28217	110~21870	
EDAc-MPs (time 2)†			0.267
mean±SD	2315.71 ± 4803.61	3237.34 ± 3474.93	
median	11026	12807.5	
interquartile range	7757.25~20321	6660.25~24240.25	
range	1239~53748	122~10827	
EDAp-MPs (time 2)†			0.200
mean±SD	10912.26 ± 5543.11	12782.32 ± 8648.35	
median	35472	61596.5	
interquartile range	8231.25~76459.75	24945.5~124349.5	
range	568~425339	48~662081	
PDAc-MPs (time 3)‡			0.009
mean±SD	163110.26 ± 250042.10	55555.49 ± 76172.00	
median	16907	8565.5	
interquartile range	10666.25~25465.25	4527.75~13739.75	
range	1050~66499	723~45969	
PDAp-MPs (time 3)‡			0.013
mean±SD	31602.12 ± 43567.74	13907.85 ± 13973.76	
median	5121	1158	
interquartile range	2212.5~13313	518~2915.25	
range	471~127265	99~8049	
EDAc-MPs (time 3)‡			0.007
mean±SD	10551.19 ± 19882.08	1900.62 ± 1889.91	
median	17211	9094	
interquartile range	8945.75~32166	5765.75~16032.75	
range	1211~206185	966~81818	
EDAp-MPs (time 3)‡			<0.000
mean±SD	19717.12 ± 13296.76	9966.81 ± 7436.74	
median	110855.5	27656.5	
interquartile range	492545~221313.75	9829.5~70369.5	
range	4831~1499326	21~465579	
ΔEDAp-MPs			0.003
Increase	64.3%(27)	36.2%(34)	
Decrease	35.7%(15)	63.8%(60)	
ΔEDAc-MPs			<0.0001
Increase	85.7%(36)	44.7%(42)	
Decrease	14.3%(6)	55.6%(52)	
ΔPDAp-MPs			0.036
Increase	50%(21)	30.9%(29)	
Decrease	50%(21)	69.1%(65)	
ΔPDAc-MPs			0.005
Increase	64.3%(27)	37.2%(35)	
Decrease	35.7%(15)	62.8%(59)	

Data expressed as mean ± SD.

PDAc-MPs = platelet-derived activated microparticles; PDAp-MPs = platelet-derived apoptotic MPs; EDAc-MPs = endothelial-derived activated MPs; EDAp-MPs = endothelial-derived apoptotic MPs.

* indicated the blood sampling was performed prior to any treatment. † indicated the blood sampling was performed at the end of 1^st^ month after pharmacological intervention. ‡ indicated the blood sample was performed at the end of the 3^rd^ month after pharmacological intervention.

ΔEDAp-MPs: EDApMPs levels that three months after treatment minus initial level

ΔEDAc-MPs: EDAcMPs levels that three months after treatment minus initial level

ΔPDAp-MPs: PDApMPs levels that three months after treatment minus initial level

ΔPDAc-MPs: PDAcMPs levels that three months after treatment minus initial level

### Comparing effects of chemotherapy and targeted therapy on circulating microparticle levels

Table 5 compares changes in circulating levels of PDAc-MPs, PDAp-MPs, EDAc-MPs, EDAp-MPs in the patients that received chemotherapy or targeted therapy. The lung cancer patients with EGFR mutant type were treated with EGFR TKI agents and those with EGFR wildtype subgroup were treated with chemotherapy based on guideline recommendations. The flow cytometric analysis showed that the circulating levels of the four types of MPs were similar between the chemotherapy and targeted therapy patients prior to and at the end of the first month after pharmacological therapy. However, at the end of the third month after pharmacological therapy, all the four types of MPs were significantly lower in patients that received targeted therapy compared to those that received chemotherapy. (Table 5)

**Table 5:** Serial changes of circulating microparticles between chemotherapy and target therapy patients

Variables	Chemotherapy (n=65)	Target therapy (n=71)	P-value
PDAc-MPs (time 1)*	223983.58 ± 1022792.58	149054.89 ± 224562.98	0.548
PDAp-MPs (time 1)*	44645.09 ± 136502.00	18485.23 ± 13633.03	0.129
EDAc-MPs (time 1)*	9618.75 ± 46275.14	9478.30 ± 12246.30	0.417
EDAp-MPs (time 1)*	42145.84 ± 15114.76	13461.90 ± 10052.02	0.132
PDAc-MPs (time 2)†	73103.35 ± 109872.19	93987.94 ± 91086.41	0.228
PDAp-MPs (time 2)†	16885.72 ± 17225.25	17386.25 ± 14031.42	0.852
EDAc-MPs (time 2)†	2613.71 ± 4444.46	3263.08 ± 3412.56	0.339
EDAp-MPs (time 2)†	11468.14 ± 7389.82	12879.21 ± 8241.49	0.297
PDAc-MPs (time 3)‡	119051.69 ± 250550.43	61049.11 ± 83443.71	0.034
PDAp-MPs (time 3)‡	24724.68 ± 36663.15	14472.15 ± 14869.75	0.039
EDAc-MPs (time 3)‡	7070.12 ± 16525.59	2285.21 ± 2554.22	0.024
EDAp-MPs (time 3)‡	15424.49 ± 12684.86	10738.13 ± 7661.49	0.011

Data expressed as mean ± SD.

PDAc-MPs = platelet-derived activated microparticles; PDAp-MPs = platelet-derived apoptotic MPs; EDAc-MPs = endothelial-derived activated MPs; EDAp-MPs = endothelial-derived apoptotic MPs.

* indicated the blood sampling was performed prior to any treatment. † indicated the blood sampling was performed at the end of 1^st^ month after pharmacological intervention. ‡ indicated the blood sample was performed at the end of the 3^rd^ month after pharmacological intervention.

### Circulating levels of microparticles in one-year survivors and non-survivors

**Figure 3:**
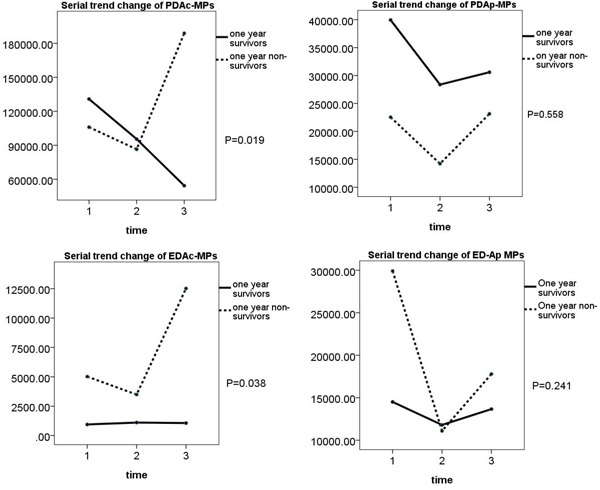
Comparison of changes in levels of the four types of microparticles in one-year survivors and non-survivors Changes in levels of **(A)** PDAc-MPs (p=0.019), **(B)** PDAp-MPs (p=0.558), **(C)** EDAc-MPs (p=0.038), **(D)** EDAp-MPs (p=0.241 in one-year survivor and non-survivor groups.

We observed that the four types of circulating MPs did not differ between 1-year survivors and non-survivors prior to and at one month time intervals after pharmacological intervention. However, at the end of the third month after pharmacological intervention, except for EDAc-MPs, the other three types of MPs were significantly higher in the one-year non-survivors than in one-year survivors. Additionally, PDAc-MPs and EDAc-MPs levels were significantly higher in 1-year non-survivors than in 1-year survivors. These findings suggest that the circulating MPs can serve as 1-year prognostic predictors in advanced stage NSCLC patients. (Table 6 and Figure 3)

**Table 6:** Serial changes of circulating microparticles between one year survivors and on year non-survivors

Variables	One year non-survivors (n=34)	One year survivors (n=102)	P-value
PDAc-MPs (time 1)*	106012.59 ± 119401.35	211151.00 ± 832025.21	0.465
PDAp-MPs (time 1)*	22543.09 ± 19448.73	33803.11 ± 109590.41	0.553
EDAc-MPs (time 1)*	5019.29 ± 7981.37	7921.78 ± 38015.19	0.660
EDAp-MPs (time 1)*	29914.29 ± 68885.70	26256.79 ± 115164.17	0.862
PDAc-MPs (time 2)†	86471.00 ± 96982.29	83184.78 ± 102325.18	0.334
PDAp-MPs (time 2)†	14217.09 ± 9419.38	18123.68 ± 17079.45	0.486
EDAc-MPs (time 2)†	3487.41 ± 5622.06	2774.49 ± 3205.34	0.098
EDAp-MPs (time 2)†	11096.35 ± 5378.55	12574.28 ± 8505.54	0.870
PDAc-MPs (time 3)‡	188877.00 ± 267863.35	55402.25 ± 78140.98	0.009
PDAp-MPs (time 3)‡	23141.74 ± 38386.77	18115.76 ± 23505.10	0.007
EDAc-MPs (time 3)‡	12522.74 ± 21674.66	1921.91 ± 1842.74	0.365
EDAp-MPs (time 3)‡	17790.21 ± 12573.57	11373.84 ± 9379.33	0.007

Data expressed as mean ± SD.

PDAc-MPs = platelet-derived activated microparticles; PDAp-MPs = platelet-derived apoptotic MPs; EDAc-MPs = endothelial-derived activated MPs; EDAp-MPs = endothelial-derived apoptotic MPs.

* indicated the blood sampling was performed prior to any treatment. † indicated the blood sampling was performed at the end of 1^st^ month after pharmacological intervention. ‡ indicated the blood sample was performed at the end of the 3^rd^ month after pharmacological intervention.

### Receiver operating characteristic (ROC) plot of circulating levels of MPs and CEA in NSCLC patients with progressive and non-progressive disease

**Figure 4:**
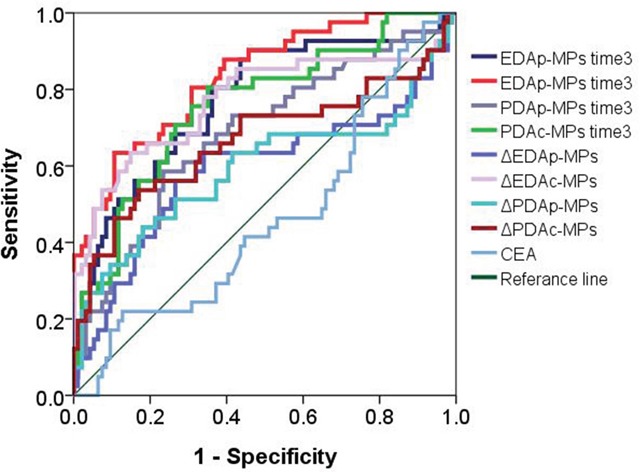
Receiver operating characteristic (ROC) plot showing circulating levels of microparticles (MPs) in progressive and non-progressive NSCLC

Next, we examined if the circulating levels of the four types of MPs and CEA could predict progressive disease in NSCLC patients. As shown in Figure 4 and Table 7, ROC curve analysis showed that the circulating levels of EDAp-MPs, EDAc-MPs, PDAp-MPs and PDAc-MPs were greater than 10468.5 counts/ml, 3557 counts/ml, 15055 counts/ml and 62700.5 counts/ml, respectively at the 3^rd^ month after therapy. These data showed that the 4 types of MPs had high sensitivity and specificity and were good prognostic predictors for advanced stage NSCLC. Further, multivariate logistic regression analysis showed that the EGFR mutant, levels of EDAc-MPs at 3^rd^ month after therapy and the ΔPDAp-MPs and ΔPDAc-MPs were independent prognostic predictors in NSCLC patients (Table 8).

**Table 7:** Value of four types of microparticles for predicting progressive disease of NSCLC patients

Variables	AUC	P value	95% CI
EDAp-MPs (time 3)	0.805	<0.000	0.680~0.861
EDAc-MPs (time 3)	0.659	<0.000	0.760~0.907
PDAp-MPs (time 3)	0.610	0.001	0.584~0.787
PDAc-MPs (time 3)	0.707	<0.000	0.667~0.847
ΔEDAp-MPs	0.590	0.098	0.471~0.708
ΔEDAc-MPs	0.771	<0.000	0.670~0.872
ΔPDAp-MPs	0.594	0.083	0.474~0.714
ΔPDAc-MPs	0.667	0.002	0.553~0.781
CEA	0.460	0.464	0.354~0.567
**Variables**	**Cut-off value**	**Sensitivity**	**Specificity**
EDAp-MPs (time 3)	10468.5	0.805	0.638
EDAc-MPs (time 3)	3557	0.659	0.840
PDAp-MPs (time 3)	15055	0.610	0.713
PDAc-MPs (time 3)	62700.5	0.707	0.734
ΔEDAp-MPs	+196	0.634	0.660
ΔEDAc-MPs	+267	0.829	0.596
ΔPDAp-MPs	+918.5	0.512	0.734
ΔPDAc-MPs	+2448	0.634	0.670
ΔCEA	+6.37	0.463	0.468

NSCLC = non small cell lung cancer; AUC = area under the curve; CI = confidence interval; NSCLC = non small cell lung cancer; PDAc-MPs = platelet-derived activated microparticles; PDAp-MPs = platelet-derived apoptotic microparticles; EDAc-MPs = endothelial derived activated microparticles; EDAp-MPs = endothelial-derived apoptotic microparticles.

ΔEDAp-MPs: EDApMPs levels that three months after treatment minus initial level

ΔEDAc-MPs: EDAcMPs levels that three months after treatment minus initial level

ΔPDAp-MPs: PDApMPs levels that three months after treatment minus initial level

ΔPDAc-MPs: PDAcMPs levels that three months after treatment minus initial level

**Table 8:** Predictors of progressive disease in non-small cell lung cancer patients by multivariate logistic regression analysis

Variable	Comparison	OR^b^ (95%CI^c^)	P-value
Body height	Per 1 unit decrease	1.179(0.009~1.387)	0.074
Adrenal gland metastasis	Yes vs. No	0.007(0.001~69.783)	0.290
CEA	Disease control vs. Progression	0.999(0.998~1.001)	0.464
Performance status	2 vs. 0&1	0.251(0.253~1.193)	0.082
EGFR mutant	Yes vs. No	150.517(8.986~2521.118)	<0.0001
EDAp-MPs (time3)	Per 1 unit decrease	1.000(1.000~1.000)	0.412
EDAc-MPs (time3)	Per 1 unit decrease	1.001(1.000~1.002)	0.022
PDAp-MPs (time3)	Per 1 unit decrease	1.000(1.000~1.000)	0.327
PDAc-MPs (time3)	Per 1 unit decrease	1.000(1.000~1.000)	0.887
ΔEDAp-MPs	positive vs. negative	0.557(0.052~6.004)	0.630
ΔEDAc-MPs	positive vs. negative	0.285(0.033~2.468)	0.254
ΔPDAp-MPs	positive vs. negative	0.074(0.006~0.892)	0.040
ΔPDAc-MPs	positive vs. negative	12.32(1.130~134.32)	0.039

^a^ΔEDAp-MPs: EDApMPs levels that three months after treatment minus initial level

ΔEDAc-MPs: EDAcMPs levels that three months after treatment minus initial level

ΔPDAp-MPs: PDApMPs levels that three months after treatment minus initial level

ΔPDAc-MPs: PDAcMPs levels that three months after treatment minus initial level

^b^Odds ratio

^c^Confidence interval

## DISCUSSION

In this study, we determined if circulating levels of MPs could predict clinical outcomes in advanced stage NSCLC patients. We observed that circulating levels of the four types of MPs that we analyzed were higher in advanced stage NSCLC patients. The levels of EDAc-MPs, EDAp-MPs, PDAc-MPs, PDAp-MPs did not differ between DC and DP prior to therapeutic intervention. However, by the end of the third month, all the four biomarkers were significantly lower in the DC group compared with the DP group and similar to control subjects. Additionally, the positive net changes of four types of microparticles (i.e., ΔEDAp-MPs, ΔEDAc-MPs, ΔPDAp-MPs and ΔPDAc-MPs) between 3^rd^ month and baseline were strongly associated with DP. Furthermore, ROC curve identified that the absolute values at three months and the relative changes of MP values over time (i.e., ΔEDAp-MPs, ΔEDAc-MPs, ΔPDAp-MPs and ΔPDAc-MPs) had notably higher sensitivity and specificity than that of the CEA level for predicting the prognostic outcomes. Moreover, multivariate logistic regression analysis exhibited that the EGFR mutant, EDAc-MPs level at 3^rd^ month and the net change between baseline and 3^rd^ month (i.e., ΔPDAp-MPs and ΔPDAc-MPs) were also independently predictive of DP in NSCLC patients. Accordingly, these suggested that these four types of circulating MPs may be useful biomarkers for predicting prognostic outcomes in LC patients.

This study also showed that the circulating levels EDAc-MPs, EDAp-MPs, PDAc-MPs were higher in study patients prior to receiving treatment than in control subjects and were consistent with previous studies [18–20]. Most importantly, the circulating levels of the four types of MPs in the DC group were significantly lower and comparable to the control subjects at end of the third month in the DC group than in the DP group. Furthermore, the circulating levels of MPs independently predicted the one-year prognostic clinical outcome in the advanced stage NSCLC patients. Thus, our findings highlight that serial measurement of circulating MPs can predict therapeutic response and prognostic outcomes in advanced NSCLC patients, especially when considering the cost and effectiveness of target therapy.

An association between aberrant EGFR mutation activity and better prognostic outcomes has been reported previously [40] [18–20]. Additionally, poor performance status and poorer prognostic outcomes were associated according to previous studies [41] [18–20]. We demonstrated that the poor performance status was significantly higher in the DP group, whereas EGFR mutations were significantly lower in the DP group.

This study has limitations. First, the sample size of this cohort study was relatively small. Therefore, a new clinical trial with larger sample size is needed to validate that circulating MP levels can predict long-term clinical outcomes in advanced NSCLC patients. Second, this study measured only advanced NSCLC patients. Therefore, the usefulness of these four biomarkers in small cell lung cancer or other types of cancer patients is not known.

In conclusion, we demonstrated that serial measurement of the circulating levels of EDAc-MPs, EDAp-MPs, PDAc-MPs, PDAp-MPs predicted prognostic outcomes in advanced NSCLC patients.

## MATERIALS AND METHODS

### NSCLC patient enrollment and therapeutic strategies

Patient enrollment, data collection, classification of advanced stage NSCLC and therapeutic strategies were according to our previous studies [18–20]. We assessed images and pathological findings of all patients who received evaluation or treatment for LC at Kaohsiung Chang Gung Memorial Hospital. We determined the eligibility of patients for interventions including surgery, adjunctive or palliative chemotherapy, irradiation therapy and/or target therapy based on the AJCC cancer staging criteria, 7^th^ edition [39]. Based on the radiological findings, we categorized LC patients into stages I, II, IIIA, IIIB, and IV according to AJCC cancer staging criteria, 7^th^ edition [42]. Patients with stage IIIB or stage IV NSCLC were categorized as advanced stage NSCLC. All the patients were enrolled for further evaluation, blood sampling and treatment in the outpatient department or upon hospital admission.

Detailed in-hospital and follow-up data including age, gender, chest x-ray findings, computed tomography, fibro-bronchoscopic findings, bone scans or ultrasound studies, other image findings, histological, pathological and laboratory findings were collected prospectively and entered into a computer database for analyses.

Informed consent was obtained from all patients and control subjects enrolled in the study. The study protocol was approved by the Institutional Review Committee on Human Research at Kaohsiung Chang Gung Memorial Hospital (IRB number: 100-1024B). The clinical investigations were conducted according to the principles outlined in the Declaration of Helsinki.

To circumvent adverse influences on measurement of circulating level of MPs, patients with one or more of the following criteria were excluded based on our previous studies [18–20]: (1) recent surgery or trauma during the preceding 2 months;(2) refusal to participate in the study; (3) other co-existent or history of malignances; (4) severe organ disease other than LC like chronic kidney disease (CKD > stage III), liver cirrhosis, hematologic disorders, congestive heart failure; (5) current use of anti-platelet agents;(6) history of febrile disorders; (7) acute or chronic inflammatory disease other than LC during the study period; or (8) a history of autoimmune diseases with or without immunosuppressive therapy.

A total of 1418 NSCLC patients were screened at Kaohsiung Chang Gung Memorial Hospital from March 2012 to January 2015. Among them, 1106 (78%) were advanced NSCLC patients. For the purpose of the study, only patients with advanced NSCLC without prior treatment were considered. Among the 1,418 patients (including NSCLC and other type of lung cancer), 1145 patients did not fit the enrolment criteria and were excluded from the study. Additionally, 35 patients were excluded due to the aforementioned reasons. Finally, 136 patients who were diagnosed with advanced stage NSCLC between March 2012 and January 2015 were prospectively enrolled in this study [stage IIIB, 22.1% (30); stage IV, 77.9% (106)] (Table 3). These 136 patients were further divided into disease controlled (DC) group (n=94) and disease progression (DP) group (n=42).

### Flow cytometry analysis of circulating microparticles

**Figure 5:**
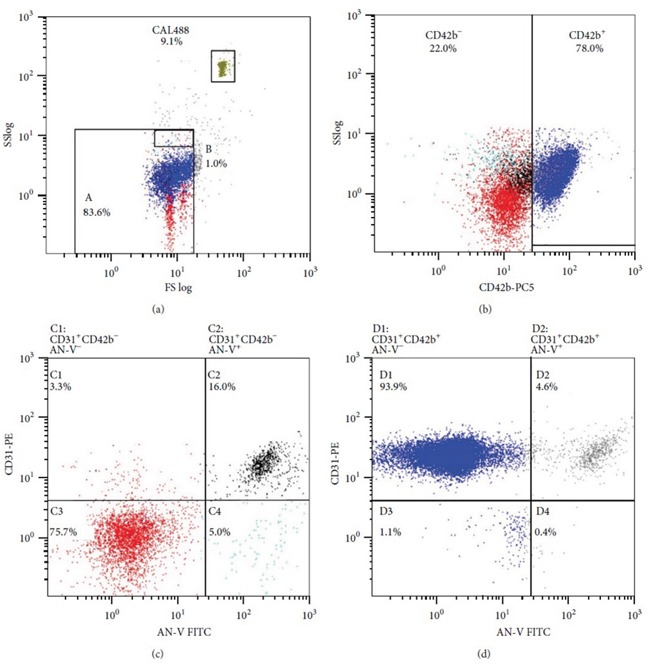
Flow cytometric analysis Representative FACS plots showing the four different types of microparticles.

As shown in Figure 5, the circulating MPs were categorized as (1) platelet-derived activated MPs (PDAc-MPs; CD31^+^ CD42b^+^ AN^-^V^-^); (2) platelet-derived apoptotic MPs (PDAp-MPs; CD31^+^ CD42b^+^ AN^-^V^+^); (3) endothelial-derived activated MPs (EDAc-MPs; CD31^+^ CD42b^-^ AN^-^V^-^); and (4) endothelial-derived apoptotic MPs (EDAp-MPs; CD31^+^ CD42b^-^ AN^-^V^+^) based on a previous study [43] with some modifications [18–20].

### Blood sample collection and processing

To determine circulating levels of MPs in advanced stage NSCLC patients, blood samples were collected at 9:00 am prior to and at the end of the first and third month after therapeutic interventions according to previously published protocol [18–20]. Additionally, blood samples were also collected at 9:00 am once from control subjects.

For flow cytometry, peripheral blood was collected in acid citrate dextrose (ACD) vacutainer tubes. Platelet-rich plasma was prepared by centrifuging 1.5ml peripheral blood at 2500 g at 4°C for 15 min without acceleration. Then, 250μl plasma samples were thawed and centrifuged at 19,800 g for 10 min at 4°C, and then collected for analyzing MPs smaller than 1.0μm.

Size calibration was conducted with 1.0μm beads (Invitrogen, Carlsbad, CA). The MP pellet was re-suspended with 150μl of AnnexinV binding buffer (BD Biosciences). All buffers were sterile-filtered with a 0.2μm filter. Then, 100μl MPs were incubated in a TruCOUNT tube (BD Biosciences) with the following fluorescent monoclonal antibodies: (1) phycoerythrin (PE)-conjugated anti-CD31 (BD Biosciences); (2) fluorescein isothiocyanate–conjugated anti-AnnexinV (BD Biosciences) and; (3) phycoerythrin-Cy5 (PE-Cy5)-conjugated anti-CD42b (BD Biosciences). The samples were incubated in the dark for 15 min at room temperature followed by addition of 400μl AnnexinV binding buffer and then analyzed in a FC500 flow cytometer (Beckman Coulter). The absolute count of MPs was measured by setting up the FACS machine with TruCount beads at 10,000 events. Additionally, white blood cell (WBC) counts, biochemistry and electrolyte levels were analyzed by standard laboratory methods in our hospital.

### Disease classification

Change in tumor burden was assessed to determine tumor response to adjunctive therapy [18–20]. The chest computed tomography (CT) scans were routinely performed at baseline and at 12 week intervals after adjunctive therapy to determine the status of the disease. The tumor measurement was based on the current guidelines of Response Evaluation Criteria in Solid Tumors (RECIST) including complete response, partial response, stable disease and progressive disease [39]. Accordingly, we categorized the disease status as disease-controlled (DC) or disease-progressed (DP). The DC status was determined at the 3rd month after the treatment and defined as disease with regression with complete response, partial response or stable disease. On the other hand, the DP was defined as disease unresponsive to therapy with a growing tumor or metastasis after complete course of treatment. To elucidate if the changes in MPs over time-course of treatment predicted outcomes, the relative levels (initial vs. third month) of the four types of microparticles were analyzed. The differences in the four types of microparticles were designated as ΔEDAp-MPs, ΔEDAc-MPs, ΔPDAp-MPs and ΔPDAc-MPs, respectively.

### Statistical analysis

Data were expressed as means ± standard deviation (SD). Continuous variables were analyzed by independent t tests and categorical variables were analyzed by the chi-square test. To determine outcomes, we compared levels of MPs three months after treatment with the initial levels. All variables were considered as risk factors with a *P* < 0.10 in univariate analysis and were further analyzed by the multivariate logistic regression analysis to identify the independent factors that predict progressive disease. Receiver operating characteristic (ROC) curves were plotted and the area under the curve and CEA levels were compared for the four types of MPs. The cutoff value of MPs for predicting progressive disease in NSCLC patients was according to ROC curves. Results were presented as absolute numbers (percentage) or mean ± SD as well as medians, interquartile ranges and ranges for the various MP results. A two-tailed P value of less than 0.05 was considered statistically significant. Statistical analysis was performed using SPSS statistical software for Windows version 13 (SPSS for Windows, version 13; SPSS Inc., IL).

## Abbreviations

ACD: acid citrate dextrose
AJCC: American joint committee on cancer
ALK: anaplastic lymphoma kinase
BD: binding buffer
CAD: coronary artery disease;
CEA: carcinoembryonic antigen
CKD: chronic kidney disease
COPD: chronic obstructive pulmonary disease
CT: computed tomography
DC: disease control
DP: disease progression
EDAc-MPs: endothelial-derived activated MPs
EDAp-MPs: endothelial-derived apoptotic MPs
EGFR: epidermal growth factor receptor
GOT: glutamic oxaloacetic transaminase
GPT: glutamic pyruvic transaminase
LC: lung cancer
MPs: microparticles
NSCLC: non-small cell lung cancer
PDAc-MPs: platelet-derived activated MPs
PDAp-MPs: platelet-derived apoptotic MPs
RBC: red blood cell
RECIST: Response Evaluation Criteria in Solid Tumors
ROC: Receiver operating characteristic
SCLC: small cell lung cancer
SD: standard deviation
TKI: tyrosine kinase inhibitor
WBC: white blood cell.
